# Impact of Cardiac Surgery Scar on Heart Rupture Following a Fall from Height

**DOI:** 10.3390/diagnostics14222472

**Published:** 2024-11-05

**Authors:** Gabriele Napoletano, Biancamaria Treves, Lina De Paola, Fabio Del Duca, Alessandro Ghamlouch, Paola Frati, Aniello Maiese

**Affiliations:** Department of Anatomical, Histological, Forensic and Orthopedic Sciences, Sapienza University of Rome, Viale Regina Elena 336, 00161 Rome, Italy; biancamaria.treves@uniroma1.it (B.T.); lina.depaola@uniroma1.it (L.D.P.); fabio.delduca@uniroma1.it (F.D.D.); alessandro.ghamlouch@uniroma1.it (A.G.); paola.frati@uniroma1.it (P.F.); aniellomaiese@msn.com (A.M.)

**Keywords:** forensic pathology, cardiopathology, cardiac post-surgery, legal medicine, cause of death, autopsy, post-mortem

## Abstract

Death from falls accounts for a significant number of injuries and fatalities globally, often linked to suicides, workplace accidents, or substance abuse, and rarely to homicidal causes. Injuries from falls vary based on height, impact point, and surface struck, with severe trauma often seen, including visceral ruptures, organ lacerations, and complex fractures. Even minimal external injuries can mask severe internal damage, such as multiple organ ruptures, organ tears, and large vessel lacerations. Blunt cardiac injuries, which occur in 5% to 50% of falls, are significant, especially in falls over 6 m. In 70% of the cases, cardiac rupture is observed at the level of the posterior wall of the heart and occurs due to a contusive action on the heart during the diastolic filling phase. We report a case of a 29-year-old man (weight 95 kg) who died from an 11-meter fall. He had a history of cardiac surgery for the transposition of the great vessels, and an autopsy revealed extensive cardiac rupture, likely worsened by fibrotic adhesions anchoring the heart to the pericardium. Toxicological investigations on peripheral blood showed BAC > 2.58 g/L. Heart scars, macro- and microscopically as a deposit of fibrous tissue, due to previous surgery, may have contributed to the extent of the lesion, suggesting the need for further study on post-operative tissue changes and their effects on trauma from falls.

**Figure 1:**
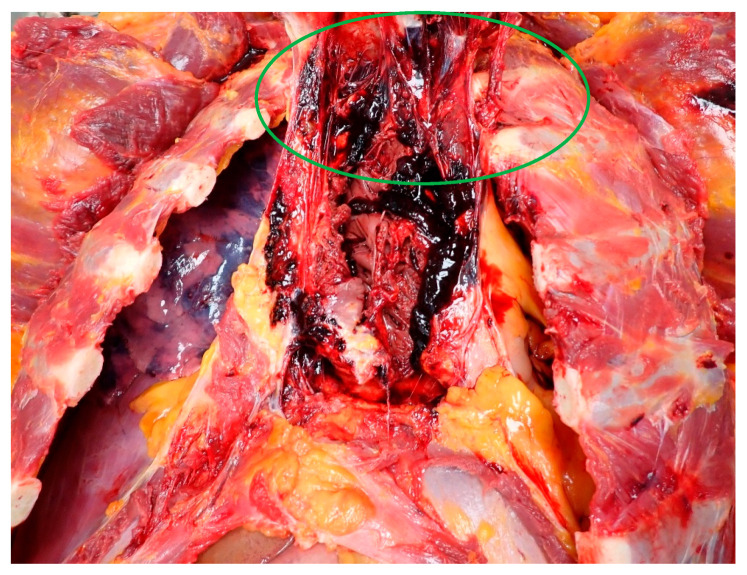
Forensic autopsy revealed contusions of the parasternal muscles as well as left-sided rib fractures, heart rupture with extensive retrosternal fibrosis (green circle), and a left hemothorax of 2000 mL. Examination of the heart showed extensive injury to the right cardiac wall with infiltration of blood along the edges and the accumulation of blood clots within the cardiac chambers. Additional findings included bronchopulmonary contusions, liver lacerations, and lacerations of the left renal capsule [1].

**Figure 2:**
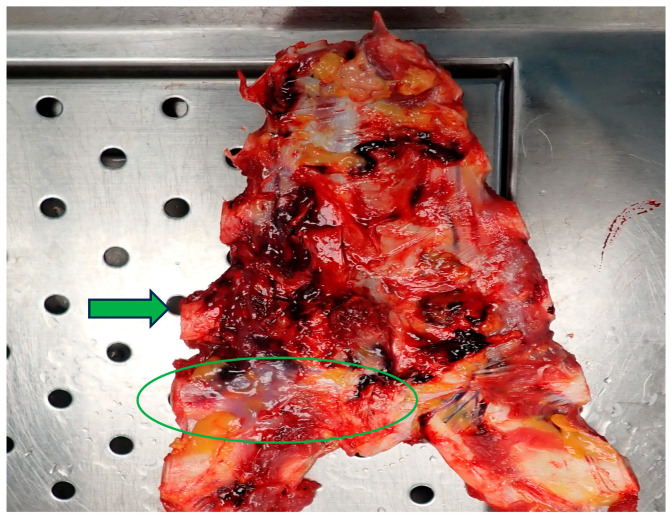
The broken anterior wall of the heart with the anterior surface of the atrium and ventricle was fixed to the retrosternal tissues (green arrow) due to a post-surgical scar (green circle). Heart rupture from a fall occurs due to rapid vertical deceleration and the impact forces acting on the organ. A study conducted on 307 cases of fatal falls revealed that 53% had heart rupture, and these injuries were more common among the victims aged 60 years or older and those falling from greater heights [2]. From a physio-pathological perspective, the mechanism of non-penetrating heart rupture depends on the pendulum-like shape of the organ. Upon impact with the ground, particularly on the anterior chest, the heart can be compressed between the sternum and the vertebral column, resulting in injuries predominantly involving the full thickness of the heart [3,4,5]. During frontal impact from a fall, the right atrium and right ventricle are the parts of the heart most likely to rupture compared to the left ventricle and left atrium, which are more commonly injured in vehicular trauma [5]. In the cases of the contusive rupture of the cardiac chambers, death occurs in 80% of the cases despite hospital arrival [6,7].

**Figure 3:**
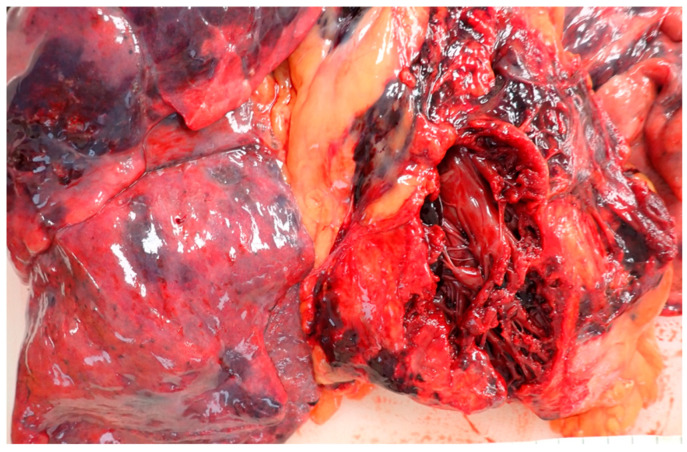
The right anterior wall was completely avulsed and adhered to the overlying retrosternal tissues. The laceration, with dark red margins, originated from the lateral edge of the right atrium, extended towards the adjacent ventricle and the cardiac apex, and then ascended along the interventricular septum towards the aortic ostium. Its extension was 11 × 7 cm. The case presented can be classified according to the American Association for the Surgery of Trauma as a Grade VI cardiac injury, which involves a “*penetrating wound producing more than 50% tissue loss of a chamber*” [8].

**Figure 4:**
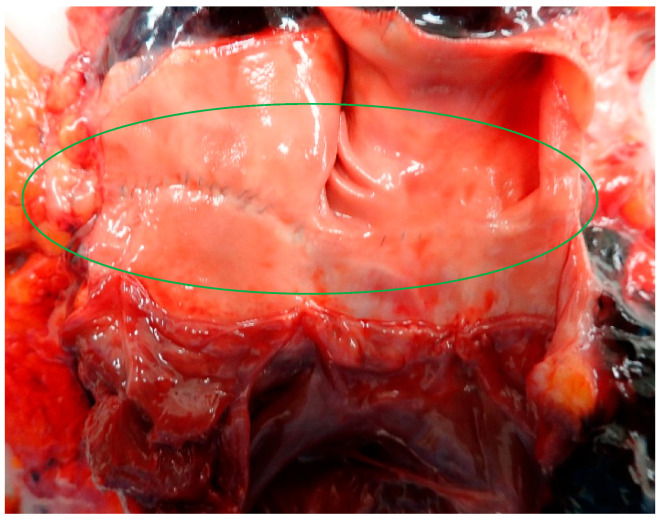
On the ascending aorta and the pulmonary artery, there were well-healed surgical scars with green sutures indicative of previous corrective surgery for the transposition of great arteries (green circle). Blunt Traumatic cardiac (BTC) ruptures (rupture of the mitral valve, endocardium, and papillary muscles) have also been described at heights less than 15 m [9,10], However, these were found to be localized injuries and of smaller size compared to those observed in the patient with retrosternal scars. A study conducted on 190 cases of BTC rupture highlighted that the majority of transmural atrial and ventricular injuries were located on the posterior wall of the organ near the vena cavae [11] and not on the anterior surface of the organ. Although Casali et al. [2] highlight an increase in heart rupture from falls from significant heights such as 12 m, this study does not report the extent and location of the cardiac injuries. These aspects have been observed by Turk et al. [12], who reported cardiac injuries approximately 1 cm in size from heights less than 15 m. This suggests that injuries of several centimeters or the total avulsion of the right atrium and ventricle from heights less than 15 m could be facilitated by anatomical and structural factors (e.g., retrosternal fibrosis). Therefore, the atypical pattern of injury, characterized by the avulsion of the atrial and ventricular walls of the heart, may suggest a stretching mechanism. Such a mechanism would be caused by the retrosternal adhesions that exerted traction on the organ during the expansion phase of the chest cage following the BTC rupture of the heart due to impact with the ground.

**Figure 5:**
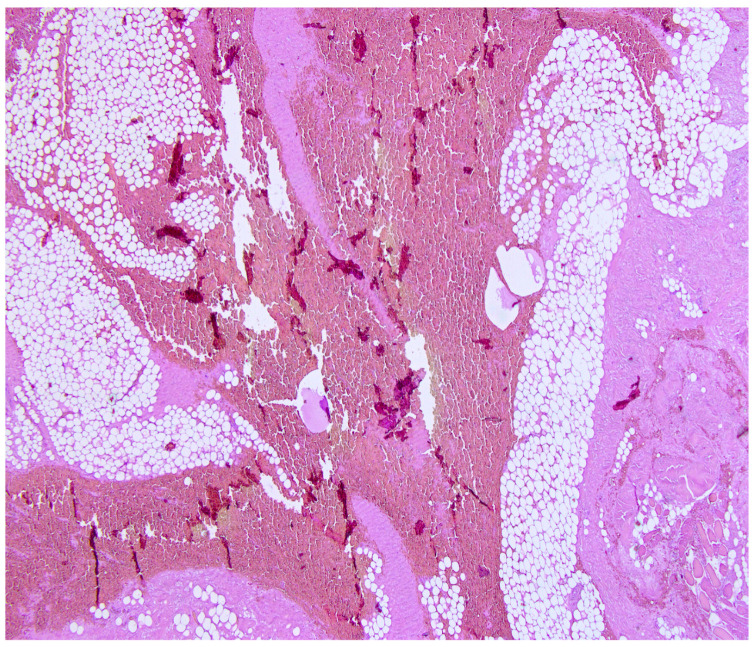
Microscopic examination of scar tissue—obtained using a Leica TCS SPE microscope, Cambridge, UK—using traditional hematoxylin and eosin (H&E) staining revealed a marked thickening of the hemorrhagic pericardium with strong adhesions to the underlying epicardium, the overlying parasternal intercostal striated muscle, and retrosternal fat and interstitial hemorrhages [13].

**Figure 6:**
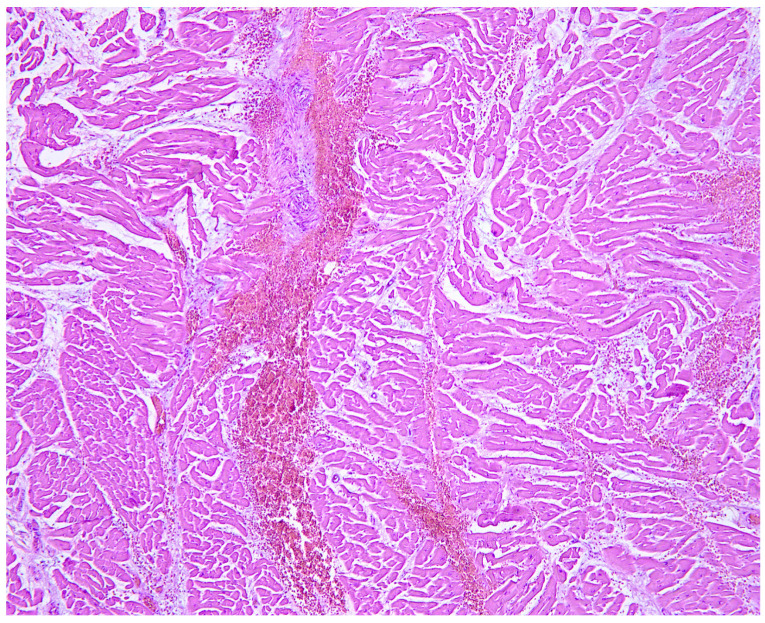
Myocardial samples showed extensive interstitial and perivascular hemorrhages.

## References

[1] Mlayeh S., Ben Abderrahim S., Haggui F., Ghzel R., Jedidi M. (2021). Deadly Falls into Wells: A Retrospective Study of 72 Autopsy Cases from Kairouan, Tunisia. J. Forensic Sci..

[2] Casali M.B., Battistini A., Blandino A., Cattaneo C. (2014). The Injury Pattern in Fatal Suicidal Falls from a Height: An Examination of 307 Cases. Forensic Sci. Int..

[3] Baldwin D., Chow K.L., Mashbari H., Omi E., Lee J.K. (2018). Case Reports of Atrial and Pericardial Rupture from Blunt Cardiac Trauma. J. Cardiothorac. Surg..

[4] Sharma M.D., Gupta N., Rajkumar T., Sharma A. (2022). Cardiac Rupture Due to a Fall from Height: The ‘Water Hammer’ Effect. Aerosp. Med. Hum. Perform..

[5] Patel K.M., Kumar N.S., Desai R.G., Mitrev L., Trivedi K., Krishnan S. (2022). Blunt Trauma to the Heart: A Review of Pathophysiology and Current Management. J. Cardiothorac. Vasc. Anesth..

[6] Teixeira P.G.R., Inaba K., Oncel D., Dubose J., Chan L., Rhee P., Salim A., Browder T., Brown C., Demetriades D. (2009). Blunt Cardiac Rupture: A 5-Year NTDB Analysis. J. Trauma.

[7] Muramatsu H., Umino K., Masuda H., Ishizawa F., Sugano Y., Honda K. (2019). Severe Cardiac Rupture by Only One Blow to the Chest in a Young Boy: An Autopsy Case. J. Forensic Sci..

[8] Blunt Cardiac Injury. https://www.aast.org/resources-detail/blunt-cardiac-injury.

[9] Türk E.E., Tsokos M. (2004). Pathologic Features of Fatal Falls from Height. Am. J. Forensic Med. Pathol..

[10] Maiese A., Scopetti M., Santurro A., La Russa R., Manetti F., D’Errico S., de Matteis A., Cingolani M., Neri M., Pinchi E. (2020). Corpse Dismemberment: A Case Series. Solving the Puzzle through an Integrated Multidisciplinary Approach. J. Forensic Leg. Med..

[11] Turan A.A., Karayel F.A., Akyildiz E., Pakis I., Uzun I., Gurpinar K., Atilmis U., Kir Z. (2010). Cardiac Injuries Caused by Blunt Trauma: An Autopsy Based Assessment of the Injury Pattern. J. Forensic Sci..

[12] Türk E.E., Tsokos M. (2004). Blunt Cardiac Trauma Caused by Fatal Falls from Height: An Autopsy-Based Assessment of the Injury Pattern. J. Trauma Acute Care Surg..

[13] Myocardial Bridging and Ecstasy: A Fatal Combination Involving a 22 Year-Old Male—PubMed. https://pubmed.ncbi.nlm.nih.gov/27394983/.

